# The use of Ambroxol for the treatment of Gaucher disease: A systematic review

**DOI:** 10.1002/jha2.852

**Published:** 2024-01-30

**Authors:** Diego Agustín Abelleyra Lastoria, Simranjeet Grewal, Derralynn Hughes

**Affiliations:** ^1^ Institute for Medical and Biomedical Education St. George's, University of London London UK; ^2^ Lysosomal Storage Disorders Unit Royal Free London NHS Foundation Trust University College London London UK

**Keywords:** Ambroxol, Gaucher disease, systematic review

## Abstract

Gaucher disease (GD) is a heterogeneous condition requiring tailored treatment approaches. The aim of this systematic review was to synthesise and evaluate current evidence pertaining to the use of Ambroxol for the treatment of GD. Published and unpublished literature databases, conference proceedings and the reference lists of included studies were searched until 23 November 2023. A narrative synthesis was performed. Database search and risk of bias assessment were performed independently by two reviewers.

Twenty‐one studies (182 patients) were included. The evidence was low in quality. Variable responses to Ambroxol were observed. Response rates were 36% and 55% in two studies reporting on type 1 GD. One study found a 22% response rate in type 2 GD, whereas another study found 29% of patients with type 3 GD reported neurological improvements. No severe adverse events were reported in the literature, with mild and reversible side effects reported. Varying response rates are to be expected (29%–100%) when treating neurological manifestations. Varying degrees of symptomatic improvement for the treatment of GD were noted in the literature. Multidisciplinary team input and clinical judgement are advised to provide personalized treatment of this complex and multi‐faceted condition.

## 1 INTRODUCTION

Gaucher disease is a lysosomal storage disorder caused by β‐glucocerebrosidase deficiency. Undegraded substrates, including glucosylceramide and glucosylsphingosine, accumulate in the monocyte lineage of the reticuloendothelial system [1]. Accumulation occurs in the liver, spleen, bone marrow and central nervous system. The latter can lead to progressive neurological manifestations, resulting in neuronopathic GD [1]. Age at symptom onset and clinical course are highly variable among patients, with its pathogenesis poorly understood [2].

Gaucher disease is classically divided into three types, according to the presence or absence of central nervous system involvement [3]. Type 1 is non‐neuronopathic (GD1), and its clinical manifestations include hepatosplenomegaly, cytopaenia and bone marrow involvement, with some patients remaining asymptomatic. It comprises approximately 95% of cases in Western countries [4]. Type 2 corresponds to acute neuronopathic GD (GD2). It is the most severe and progressive form and can lead to hydrops fetalis, congenital ichthyosis, and consequent childhood death (usually before 2 years of age) [3, 5]. Type 3 Gaucher disease (GD3) is chronic in nature, and its manifestations include visceral involvement, ataxia and myoclonic epilepsy [3].

Given the clinically heterogeneous nature of GD, therapeutic approaches must be tailored to individual patient presentations [2]. Enzyme replacement therapies (ERTs) and substrate reduction therapies (SRTs) are the main therapeutic regimes used to treat GD. The former is the standard of care for symptomatic GD1 [6, 7]. Examples include alglucerase, velaglucerase alfa, taliglucerase alfa and imiglucerase [2, 8]. SRTs such as miglustat and eliglustat reduce the accumulation of glucosylceramide [9] and are also recommended for the treatment of GD1 [2]. There is no widely accepted therapy for the neurologic manifestations of GD2 and GD3 [10], with highly variable responses to treatment observed in clinical practice [2]. Enzyme replacement therapy does not cross the blood‐brain barrier but is indicated for the systemic manifestations of GD3.

Pharmacological chaperone therapies for the treatment of GD were first investigated in the 1990s [11, 12]. Of more than 860 known GBA1 variant alleles, many are translated into misfolded glucocerebrosidase (GCase) protein. The protein is designated for endoplasmic reticulum‐associated degradation in the proteasome. The proteasome is overwhelmed, resulting in the aggregation of GCase [13]. Ambroxol, an oral mucolytic available over the counter, was found to act as a pharmacological chaperone for mutant GCase when taken at a high dose in 2009 [14], binding to the misfolded GCase and facilitating its correct folding and functional recovery [5, 13].

Numerous case reports have described satisfactory outcomes in patients with GD following treatment with Ambroxol [15, 16, 17, 18, 19, 20]. However, no systematic reviews aimed at evaluating Ambroxol as a therapeutic alternative for the treatment of GD have been performed. A synthesis of current evidence may inform decisions regarding the treatment of patients with GD. Therefore, the aim of this systematic review was to synthesise and evaluate current evidence pertaining to the use of Ambroxol for the treatment of GD.

## 2 MATERIALS AND METHODS

The PRISMA 2020 checklist was satisfied in the reporting of this systematic review [21]. The protocol for this systematic review was prospectively registered (PROSPERO Registration: CRD42023426572).

### 2.1 Study eligibility

Studies were eligible if they reported on the use of Ambroxol for the treatment of patients with a confirmed diagnosis of GD (including paediatric and adult populations). Both full‐text and abstract were included. Eligible study designs were randomized controlled trials, cohort, case‐control and cross‐sectional studies, as well as case series. Both retrospective and prospective studies were eligible. Papers not reporting original data such as literature or systematic reviews were excluded, along with letters to the editor, laboratory studies and papers which did not specify the number of patients receiving Ambroxol. There was no constraint based on language, publication status or patient demographics. The eligibility assessment was performed independently by two reviewers (Diego Agustín Abelleyra Lastoria and Simranjeet Grewal). Disagreements regarding study eligibility were solved through discussion.

### 2.2 Search strategy

We searched the following electronic databases via OVID: MEDLINE, Global Health, Embase, Web of Science, Scopus and ScienceDirect. Currently, registered studies were reviewed using the databases: ISRCTN registry, the UK National Research Register Archive, the World Health Organization International Clinical Trials Registry Platform and the National Institute for Health Research Portfolio. Conference proceedings from the European Hematology Association and the American Society of Hematology were searched. The reference lists of included studies were also searched (backwards searching). Papers citing the studies included were also reviewed for eligibility (forward‐searching).

Database search and data extraction were conducted independently by two reviewers (Diego Agustín Abelleyra Lastoria and Simranjeet Grewal). Searches were conducted twice for quality assurance. The final search was completed on 23 November 2023. The following search strategy was utilized, and modified for each respective database:

Gaucher's disease OR Gaucher disease

AND

Ambroxol OR Muciclar OR Mucosolvan OR Mucobrox OR Bisolvon OR Mucol OR Lasolvan OR Mucoangin OR Surbronc OR Brontex OR Ambro OR Ambolar OR Lysopain

Deduplicate

### 2.3 Data extraction

Data extracted included baseline characteristics including number of patients, patient sex, age, study location, follow‐up duration and GD type. Outcomes extracted included baseline laboratory values/symptoms, outcomes of treatment, percentage of patients suffering from adverse events and type of adverse events reported. Quantitative pooled analysis was prevented by heterogeneous study designs, and differing treatment regimens, study populations and outcomes evaluated. Therefore, a narrative synthesis was performed.

### 2.4 Methodological appraisal

The level of evidence of the studies presented was determined with the March 2009 Oxford Centre for Evidence‐Based Medicine: Levels of Evidence (5 = lowest level of evidence, corresponding to case reports; 1a = highest level of evidence, corresponding to systematic reviews of randomised controlled trials) [22]. The Institute of Health Economics case series studies quality appraisal checklist [23] was used to assess the risk of bias of case series, and the tool for evaluating the methodological quality of case reports was used to assess the risk of bias of case reports [24]. The level of evidence and risk of bias of each included study were evaluated independently by two reviewers (Diego Agustín Abelleyra Lastoria and Simranjeet Grewal).

## 3 RESULTS

**FIGURE 1:**
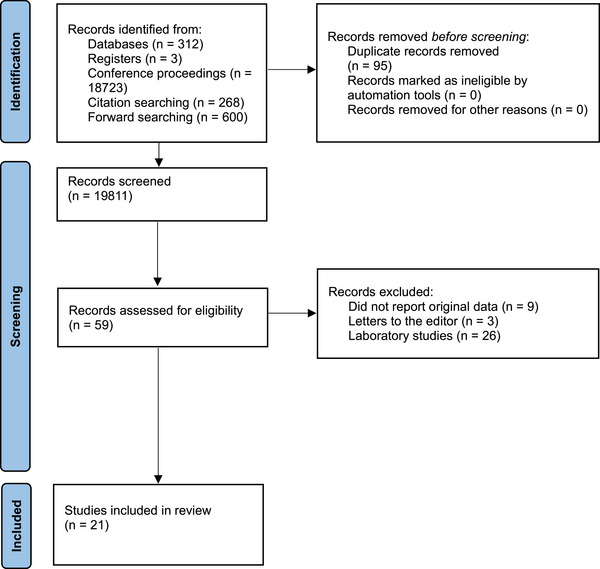
PRISMA diagram depicting the study collection process.

In total, 19,811 records were screened, of which 21 studies were eligible (Figure 1). Nine case series were included and evaluated 165 patients (Table 1). Mean patient age ranged from 4.4 months to 60.2 years. Seven studies reported patient sex (73 males, 47.7% and 80 females, 52.3%). In addition, 12 case reports were included, comprising 17 patients (eight males and nine females), with a mean age of 12.3 years (range: 7 weeks–60 years) (Table 2).

**TABLE 1:** Baseline characteristics of case series included in a systematic review.

Study	Study location	Number of patients (males, females)	Patient age	Management strategy	Follow‐up duration
Goker‐Alpan et al., 2023 [25]	Virginia, USA	12	Median: 7 months	Ambroxol 10 mM	5 years
Kim et al., 2020 [1]	Seoul, South Korea	4 (0, 4)	Mean: 17.3 (Range: 14.6 – 20.1)	Abcertin + Ambroxol (initiated at 1.5 mg/kg/day, escalated up to 27 mg/ kg/day)	4.5 years
Zimran et al., 2013 [35]	Israel	12 (4, 8)	Mean: 41.4 (Range: 24–63)	Two capsules of 75 mg of Ambroxol daily for 6 months	6 months
Istaiti et al., 2023 [39]	Israel	40 (21, 19)	Median: 52 (Range: 24 – 84)	600 mg Ambroxol per day	12 months
Istaiti et al., 2021 [28]	Jerusalem, Israel	41 (16, 25)	Median: 17 (Range: 1.5–74)	Median: Ambroxol 435 mg/day (Range: 75–1485)	Median: 19 months
Narita et al., 2016 [5]	Japan	5 (0, 5)	Mean: 18.2 (Range: 3 – 28)	25 mg/kg/day or a maximum dose of 1300 mg/day	Mean: 30 months
Lal et al., 2020 [3]	NR	5 (2, 3)	At diagnosis: Mean: 4.4 months (Range 1 week – 6 months)	Ambroxol monotherapy: 2 Ambroxol + ERT: 3	74 months
Mullin et al., 2020 [29]	London, UK	18 (15, 3) With GBA1 mutation: 8 (7, 1) Without GBA1 mutation: 10 (8, 2)	Mean: 60.2 ± 9.7 With GBA1 mutation: 56.1 ± 9,2 Without GBA1 mutation: 63.4 ± 9.2	Escalating dose of oral Ambroxol to 1.26 g per day (420 mg three times per day)	279 days
Zhan et al., 2023 [30]	Shanghai, China	28 (15, 13)	16.9 ± 15.3	Escalating dose of oral Ambroxol (mean 12.7 mg/kg/day)	2.6 years

**TABLE 2:** Baseline characteristics of case reports included in a systematic review.

Study	Study location	Patient's age, sex	Management strategy	Follow‐up duration
Balasubramaniam et al., 2023 [34]	Australia	22‐month‐old, male	Dose—NR, Duration—NR (initiation at 2 weeks), Concomitant therapies—ERT	22 months
Aries et al., 2022 [19]	Germany	3 months, female	Dose – 7.5 mg/mL was administered at a high dose of 25 mg/kg body weight per day, divided into three doses, Duration – 104 weeks of treatment, Concomitant therapies—Biweekly intravenous enzyme replacement therapy (ERT) with Imiglucerase was started at 15 months of age	104 weeks
Zhang et al., 2022 [20]	China	36 years old, male	Dose – 660 mg/day, Duration – up to 2 years, Concomitant therapies—NR	6 months
Darling et al., 2021 [33]	Spain	1 year old, twins (male and female)	Dose – NR, Duration – ∼ one year, Concomitant Therapies—enzyme replacement therapy (ERT) with intravenous imiglucerase was initiated by age 20 months, Levodopa at age 6	6 years
Ramadža et al., 2021 [18]	Croatia	5 years old, female 7 weeks, male	Dose – 25 mg/kg/day in five divided doses Duration – 6 years, Concomitant therapies—ERT with imiglucerase was started at the dose of 60 IU/kg every other week Dose ‐ 25 mg/kg/day divided into six doses, Duration – 7 years, Concomitant therapies—ERT with imiglucerase was commenced at the age of 3 months, at 60 mg/kg every other week	7 years
Ciana et al., 2020 [32]	Italy	14 years and 6 months, female 60 years old, male	Dose – 20 mg/kg/day in 2 doses and then, after 12 months, at 25 mg/kg/day in 3 doses, Duration – 42 months, Concomitant Therapies – ERT, sodium valproate, levetiracetam and clonazepam Dose—Maximum dose of 1300 mg/daily administered in three divided doses, Duration – 1 year, Concomitant Therapies—ERT	42 and 12 months, respectively
Chu et al., 2020 [31]	Taiwan	8 months, female	Dose—30 mg/kg/day, Duration – 10 months, Concomitant therapy—ERT	10 months
Jiang et al., 2020 [16]	China	5 years old, female	Dose—10 mg/kg/day (initially) → 15 mg/kg/day when no obvious adverse drug reactions were observed, Duration – approx. 2.5 years, Concomitant therapy—None	34 months
Pawlinski et al., 2020 [17]	Poland	38 years old, female	Dose ‐ 150 mg/day (initially) → 300 mg/day → 450 mg/day (withing 4 months of starting) → reduction to constant dose of Duration – approx. 2 years, Concomitant therapies – ERT, tramadol 200 mg/day, paracetamol 2 g/day, flupirtine 300 mg/day, local lidocaine blockades and physiotherapy	3 years
Charkhand et al., 2019 [15]	Canada	15 year old, male 21 year old, female	Dose ‐ 25 mg/kg/day, Duration – 3 years, Concomitant therapy – 60 IU/kg of imiglucerase biweekly, levetiracetam, clonazepam, felbamate, dilantin and escitalopram Dose – 25 mg/kg/day, Duration – 3 years, Concomitant therapy ‐ 60 IU/kg of imiglucerase biweekly, stiripentol, clobazam, lamotrigine and dilantin, and cannabidiol oil	3 years
Stanescu et al., 2019 [27]	NR	2 × 5 years old, male	Dose—NR, Duration – ∼ 1 year, Concomitant therapy – NR Dose—NR, Duration – ∼ 1 year, Concomitant therapy – ERT, antiepileptic therapy	1 year
Bello and Cortez, 2018 [26]	NR	2 months old, female	Dose ‐ 15 mg/kg/day, Duration – 9 months, Concomitant therapy—Imiglucerase, biweekly, at 60 units/kg increased to 120 units/kg ERT	9 months

Abbreviations: ERT, enzyme replacement therapy; NR, not reported.

### 3.1 Study quality assessment

Nine studies identified were case series (level of evidence 4). There was one non‐full text case series for which the risk of bias could not be assessed [25]. No studies reported whether outcome assessors were blinded to patient characteristics or treatment regimens, which could potentially lead to bias in the assessment of results (Table 3). The remaining 12 studies were case reports. Of these, two were non‐full‐text studies for which the risk of bias could not be assessed [26, 27]. The report by Ramadža et al. [18] carried a low risk of bias, with the remaining nine case reports carrying a high risk of bias (Table 3). Overall, all studies included exhibited methodological limitations pertaining to low levels of evidence and concerns regarding the risk of bias.

**TABLE 3:** Results of risk of bias assessment.

Case series quality appraisal checklist [**23**] risk of bias assessment questions	Kim et al., 2020 [**1**]	Zimran et al., 2013 [**35**]	Istaiti et al., 2023 [**39**]	Istaiti et al., 2021 [**28**]	Narita et al., 2016 [**5**]	Lal et al., 2020 [**3**]	Mullin et al., 2020 [**29**]	Zhan et al., 2023 [**30**]
Was the hypothesis/aim/objective of the study clearly stated?	Yes	Yes	Yes	Yes	Yes	Yes	Yes	Yes
Was the study conducted prospectively?	Yes	Unclear	Yes	No	Unclear	No	Yes	Unclear
Were the cases collected in more than one centre?	No	Unclear	No	Yes	Yes	Unclear	No	No
Were patients recruited consecutively?	Unclear	Unclear	Unclear	Unclear	Unclear	Unclear	Unclear	Unclear
Were the characteristics of the patients included in the study described?	Yes	No	Yes	Yes	Yes	Yes	Yes	Yes
Were the eligibility criteria (i.e., inclusion and exclusion criteria) for entry into the study clearly stated?	Yes	Partial	Yes	Yes	Yes	No	Yes	Partial
Did patients enter the study at a similar point in the disease?	Yes	Unclear	Unclear	No	Yes	Yes	Yes	Yes
Was the intervention of interest clearly described?	Yes	Yes	Yes	Yes	Yes	No	Yes	Yes
Were additional interventions (co‐interventions) clearly described?	Yes	Not applicable	Not applicable	Not applicable	Not applicable	No	Not applicable	Not applicable
Were relevant outcome measures established a priori?	Yes	Unclear	Yes	Yes	Unclear	Unclear	Yes	Unclear
Were outcome assessors blinded to the intervention that patients received?	No	Unclear	No	Unclear	No	Unclear	No	Unclear
Were the relevant outcomes measured using appropriate objective/subjective methods?	Yes	Yes	Yes	Yes	Yes	Yes	Yes	Yes
Were the statistical tests used to assess the relevant outcomes appropriate?	No	Not applicable	No	Not applicable	Yes	Not applicable	Yes	Yes
Was follow‐up long enough for important events and outcomes to occur?	Yes	Yes	Yes	Yes	Yes	Yes	Yes	Yes
Were losses to follow‐up reported?	No	No	Yes	Yes	No	Unclear	No	Yes
Did the study provide estimates of random variability in the data analysis of relevant outcomes?	Yes	No	No	Not applicable	Yes	No	Yes	Yes
Were the adverse events reported?	Yes	Yes	Yes	Yes	Yes	No	Yes	Yes
Were the conclusions of the study supported by the results?	Yes	Yes	Yes	Yes	Yes	Yes	Yes	Yes
Were both competing interests and sources of support for the study reported?	Yes	No	Yes	Yes	Yes	Yes	Yes	Yes
Risk of bias assessment	Some concerns	High	High	Some concerns	Some concerns	High	Some concerns	Some concerns

Tool for evaluating the methodological quality of case reports [**24**] risk of bias assessment questions	Balasubramaniam et al., 2023 [**34**]	Aries et al., 2022 [**19**]	Zhang et al., 2022 [**20**]	Darling et al., 2021 [**33**]	Ramadža et al., 2021 [**18**]	Ciana et al., 2020 [**32**]	Chu et al., 2020 [**31**]	Jiang et al., 2020 [**16**]
Does the patient(s) represent(s) the whole experience of the investigator (centre) or is the selection method unclear to the extent that other patients with similar presentation may not have been reported?	Unclear	Yes	Unclear	Yes	Yes	Yes	Yes	Yes
Was the exposure adequately ascertained?	Partial	Yes	Partial	Yes	Yes	Yes	Yes	Yes
Was the outcome adequately ascertained?	Partial	Yes	Partial	Partial	Yes	Partial	Partial	Yes
Were other alternative causes that may explain the observation ruled out?	No	Partial	No	No	Yes	No	No	Partial
Was there a challenge/rechallenge phenomenon?	No	No	Unclear	Partial	No	No	No	No
Was there a dose‐response effect?	No	Yes	Unclear	Unclear	Yes	No	Unclear	No
Was follow‐up long enough for outcomes to occur?	Partial	Partial	No	Partial	Yes	Partial	No	Partial
Is the case(s) described with sufficient details to allow other investigators to replicate the research or to allow practitioners to make inferences related to their own practice?	No	Yes	Partial	Partial	Yes	Partial	Partial	Yes
Risk of bias assessment	High	High	High	High	Low	High	High	High

Tool for evaluating the methodological quality of case reports [**24**] risk of bias assessment questions (continued)	Pawlinski et al., 2020 [**17**]				Charkhand et al., 2019 [**15**]			
Does the patient(s) represent(s) the whole experience of the investigator (centre) or is the selection method unclear to the extent that other patients with similar presentation may not have been reported?	Partial				Unclear			
Was the exposure adequately ascertained?	Yes				Yes			
Was the outcome adequately ascertained?	No				Partial			
Were other alternative causes that may explain the observation ruled out?	No				No			
Was there a challenge/rechallenge phenomenon?	Unclear				No			
Was there a dose‐response effect?	Unclear				No			
Was follow‐up long enough for outcomes to occur?	No				Partial			
Is the case(s) described with sufficient details to allow other investigators to replicate the research or to allow practitioners to make inferences related to their own practice?	Partial				No			
Risk of bias assessment	High				High			

### 3.2 The effect of Ambroxol on clinical outcomes

Outcomes following treatment with Ambroxol in the case series included are presented in Table 4. Goker‐Alpan et al. [25] reported on 12 infants with Type 2 GD receiving Ambroxol 10 mM. Three distinct phenotypes were observed: early onset, classical (associated with facial dysmorphism) and nonclassical. Negative response (GCase increased by 20% or less, death by 24 months, or ventilatory support) was observed in three of nine patients, whereas positive response (GCase increased by 100% or more, survival without tracheostomy beyond age 5, or walking by 15 months) was observed in two of nine. The response in the remaining seven patients was not reported.

**TABLE 4:** Outcomes following treatment with Ambroxol in case series.

Study	Type of Gaucher disease	Clinical manifestations/laboratory values	Outcomes	Rate of adverse events	Adverse events noted (number of patients experiencing these)
Goker‐Alpan et al., 2023 [25]	Type 2	Hepatosplenomegaly, thrombocytopenia. Stridor (7/11), abnormal auditory brain response (11/11) and swallowing (9/12), opisthotonus 3/11), seizures (4/ 11) abnormal liver enzymes and cholestatic jaundice (2/12). The most common GBA variant was L444P (8/12).	Negative response (GCase increased by 20% or less, death by 24 months, or ventilatory support) in three of nine. Positive response (GCase increased by 100% or more, survival without tracheostomy beyond age 5, or walking by 15 months) in two of nine.	NR	NR
Kim et al., 2020 [1]	Type 3	Three patients had myoclonus, generalized tonic‐clonic seizures and intellectual deterioration. One patient had myoclonus and intellectual deterioration. Mean baseline pre‐ERT GCase activity in peripheral leukocytes: 5.1 ± 0.6 (range, 4.7–5.8)% of the mean normal activity. mSST: 12.3 ± 6 (7–20) K‐MBI: 66.3 ± 36.2 (33–100)	At 21 mg/kg/day, the mean GCase activity in peripheral activity was 9.8 ± 3.2 (5.5–13.2)% of the mean normal activity. GCase activity increased up to 13.7 ± 4.4 (8.7–19.2)% of the mean normal activity at an ABX dose of 27 mg/kg/day.^*^ mSST at 2.5 years: 15.1 ± 6.2 (9.5–21) mSST at 4.5 years: 12.4 ± 5.3 (7–18.5) K‐MBI at 2.5 years: 51 ± 32.7 (24–88) K‐MBI at 4.5 years: 71.8 ± 30.6 (41–100)	50%	Respiratory difficulty due to an increase in mucus production (1) and transient proteinuria (1)
Zimran et al., 2013 [35]	Type 1	Mean: Body weight (kg): 66.4 Haemoglobin (gm%): 12.0 Platelets (10^3^/mm^3^): 84.4 Liver volume (cm^3^): 2214 Spleen volume (cm^3^): 1616 Chitotriosidase activity (nmol/h/mL): 5472	Mean: Body weight (kg): 66.2 Haemoglobin (gm%): 12.3 Platelets (10^3^/mm^3^): 92.4 Liver volume (cm^3^): 2203 Spleen volume (cm^3^): 1357 Chitotriosidase activity (nmol/h/mL): 5444	8.5%	Hypersensitivity reaction (mild facial rash), resulting in withdrawal (1)
Istaiti et al., 2023 [39]	Type 1	Platelet count: 173.4 × 10^3^/mm^3^ Lumbar spine T‐score: −2.3 Lyso‐Gb1 level: 231.5 ng/mL	20% or greater improvement in: Platelet count: five patients Lumbar spine T‐score: six patients Lyso‐Gb1: three patients	32.5% (of the 40 initially enrolled on treatment)	Abdominal pain/diarrhoea (2), cough (1), nausea (1), depression/nightmares (3), atypical chest pain (1), vertigo/dizziness/ headache (3), elevated liver enzymes (1), hypertension (1) and elevated Lyso‐Gb1 (2)
Istaiti et al., 2021 [28]	Type 1: 11 Type 2: 3 Type 3: 24 GBA mutation carriers with PD: 3	NR	Clinical benefits in 61%	29.3%	Minor bowel discomfort (1), cough (2), increased mucus production (2), dizziness (2), allergic reaction/skin rash/redness (1), mild proteinuria (1) and disease progression (1)
Narita et al., 2016 [5]	Type 2: 1 Type 3: 4	Haemoglobin (g/dL): 13.0 Platelets (x10^9^/L): 14.5 ACE (Units/L): 17.2 Uric acid (mg/dL): 3.9 QTc interval (ms): 402 GCase activity (nmol/mg): 8.8 CSF GlcSph (pg/mL): 175.0	Haemoglobin (g/dL): 13.3 Platelets (x10^9^/L): 14.1 ACE (Units/L): 10.3 Uric acid (mg/dL): 2.6 QTc interval (ms): 404 GCase activity (nmol/mg): 19.1^*^ CSF GlcSph (pg/mL): 142.5^*^	60%	Skin rash (1), hypouricaemia (2)
Lal et al., 2020 [3]	Type 2	Apnoea, secretions, stridor, tracheostomy, choking, hepatosplenomegaly, failure to thrive, gastroesophageal reflux disease, umbilical hernia, hypotonia/hypertonia, poor suck, myoclonus, fever, fussy, strabismus, decreased blinking and skin peeling	Two patients died (at 17 and 3 months). At the time of writing, three children receiving ERT and Ambroxol were alive (aged 74, 17 and 27 months).	NR	NR
Mullin et al., 2020 [29]	GD‐associated PD	GCase activity: ‐Blood (nmol/mg/h): 11 ‐CSF (nmol/mL/h): 0.309 GCase protein level (pMol/L): 250α‐Synuclein (pg/mL): ‐Blood: 20,793 ‐CSF: 383 Tau (pg/mL): ‐Blood: 1 ‐CSF: 206 CSF Glucosylceramide (pmol/L): 246MDS‐UPDRS score: 52.6MoCA score: 25.0NMSS score: 49.3NMSQuest score: 10.6	Day 186:GCase activity:Blood (nmol/mg/h): 12 ‐CSF (nmol/mL/h): 0.250^*^ ‐GCase protein level (pMol/L): 338^*^ α‐Synuclein (pg/mL): ‐Blood: 23,395 ‐CSF: 433^*^ Tau (pg/mL): ‐Blood: 0.8^*^ ‐CSF: 211 CSF Glucosylceramide (pmol/L): 260MDS‐UPDRS score: 63.9^*^MoCA score: 26.7NMSS score: 60.8^*^NMSQuest score: 10.8	NR	Nausea/vomiting (5), rash (3), dizziness (3), reflux (2), diarrhoea (2), memory loss (1) and rhinorrhoea (1)
Zhan et al., 2023 [30]	Type 1: 25 Type 2: 2 Type 3: 1	Haemoglobin concentration (g/dl): 10.4 Platelet count (x10^3^/μL): 69 Spleen volume (multiples of normal): 17.5 Liver volume (multiples of normal): 1.90 Chitotriosidase activity (nmol/mL/h): 14598 Glucosylsphingosine level (ng/mL): 251.3	Haemoglobin concentration (g/dl): 11.9 (*p* < 0.001)^*^ Platelet count 78 (*p* = 0.09) Spleen volume (multiples of normal): 12.3 (*p* = 0.04)^*^ Liver volume (multiples of normal): 1.50 (*p* = 0.03)^*^ Chitotriosidase activity (nmol/mL/h): 8312 (*p* = 0.001)^*^ Glucosylsphingosine level (ng/mL): 165.7 (*p* = 0.006)^*^	10.7%	Nausea (1), salivation (1), diarrhoea and rash (1)

Abbreviations: ACE, angiotensin‐converting enzyme; ABX, Ambroxol; CSF, cerebrospinal fluid; ERT, enzyme replacement; GCase, glucocerebrosidase; K‐MBI, Korean modified Barthel index; LysoGb1/GlcSph, glucosylsphingosine; MoCA, Montreal cognitive assessment; MDS‐UPDRS, Movement Disorder Society—Unified Parkinson's Disease Rating Scale; mSST, modified severity scoring tool; NMSS score, non‐motor symptom scale; NMSQuest score, non‐motor symptoms questionnaire score; NR, not reported; PD, Parkinson's disease; SRT, substrate replacement.

* Statistically significant change from baseline.

Kim et al. [1] reported on the use of Ambroxol for the treatment of patients with GD and myoclonic epilepsy. Two patients had noticeable improvements in swallowing and speech. One of these also experienced improvements in pyramidal and extrapyramidal symptoms. Two patients showed improvement in epilepsy and cerebellar tremor. Korean modified Barthel index decreased from baseline to 2.5 years (66.3 ± 36.2 to 51 ± 32.7), but this then improved at 4.5 years (71.8 ± 30.6). The Korean Wechsler Adult Intelligence Scale was to evaluate changes in intelligence. At baseline, the mean verbal comprehension index, perceptual reasoning index, working memory index, processing speed index and full‐scale intelligence quotient were 65 ± 6.8, 55 ± 10, 60 ± 6.6, 50 ± 2.1, and 52 ± 6, respectively. After 4.5 years, these were 66 ± 7.4, 52 ± 3.4, 53 ± 3.5, 50 ± 0 and 48 ± 4.1, respectively. Overall, no significant improvements in intelligence were noted after 4.5 years, in addition to no improvement in horizontal and vertical saccadic eye movements. Eye movements deteriorated in two patients and remained unchanged in the other two patients. Standing and walking balance was maintained in two patients, whilst it deteriorated in one until 2.5 years of study, and then recovered at 4.5. No recovery was observed in one patient.

Istaiti et al. [28] reported on 41 patients receiving Ambroxol for GD. Of these, 13 discontinued it (eight due to adverse events, four due to reimbursement issues and one for personal reasons). Clinical benefits were reported in 25 patients (61.0%). However, the improvements noted varied between patients. Of the 11 type 1 patients, six noted improvements (including improvement in pain and fatigue and no neurological deterioration). Only one of the three patients with type 2 GD noted neurological improvement. Of the 24 patients with type 3 GD, seven did not experience neurological deterioration, and seven noted neurological improvement. Of three GBA mutation carriers with Parkinson's disease (PD), one reported improvements in Parkinsonian symptoms.

Narita et al. [5] reported on five patients with GD suffering from myoclonus. All patients exhibited marked improvement in myoclonus and decreased Unified Myoclonus Rating Scale scores. However, the Ambroxol dose from which improvements were noted varied between patients (e.g. 9 mg/kg/day in two patients and 12 mg/kg/day in one). Two patients experienced a decrease in the frequency of seizures (9 and 15 mg/kg/day in each). One patient experienced a reduction in the duration of generalised tonic‐clonic seizures (15 mg/kg/day), and one patient experienced a reduction in frequency. The frequency of generalised convulsive status epilepticus decreased following Ambroxol treatment in two patients. The constriction rate and latency of the pupillary light reflex improved in all patients.

Lal et al. [3] reported on 23 patients with type 2 GD. Of these, five were treated with Ambroxol. Though Lal et al. reported overall outcomes in their cohort, those in patients treated with Ambroxol were not reported separately. The only exception was mortality: two patients died aged 17 and 3 months (11 and 2.5 months following diagnosis, respectively), whereas three children (receiving ERT and Ambroxol) were alive at ages 74, 17 and 27 months.

Mullin et al. [29] administered Ambroxol to 18 patients with GD‐associated PD. Improvements in Movement Disorder Society—Unified Parkinson's Disease Rating Scale and Non‐motor Symptom Scale scores at 186 days were statistically significant. Ambroxol was well tolerated, with no serious adverse effects reported. In 28 patients receiving Ambroxol for 2.6 years, Zhan et al. [30] found patients initiating treatment at a younger age experienced more rapid improvements in symptoms. Clinical improvements (including reduced fatigue, nosebleeds, petechiae and improved energy and neurologic status) were noted in 26 of 28 patients (92.9%).

Outcomes following treatment with Ambroxol in the case reports included are presented in Table 5. Of 12 reports, two did not report on clinical outcomes [20, 31], two reported no change or deterioration in symptoms [32, 33], and the remaining reported symptomatic improvements. These included resolution of respiratory compromise [34], normal neurological and cardiovascular function [19], decreased hepatosplenomegaly [26], ataxia improvement [15], analgesic effect [17] and complete symptom resolution [18]. Three studies reported adverse events occurred. These included increased mucus production, high‐pitched screaming and hyperextension in a prone position [19], soft stools [18] and mild abdominal discomfort [32].

**TABLE 5:** Outcomes following treatment with Ambroxol in case reports.

Study	Type of Gaucher disease	Clinical manifestations/laboratory values	Outcomes	Adverse events noted
Balasubramaniam et al., 2023 [34]	Type 2	Clinical manifestations: respiratory distress due to diaphragmatic splinting from hepatosplenomegaly, thrombocytopenia and anaemia Laboratory values: Beta‐glucocerebrosidase activity: 1.4 nmol/h/mg Plasma glucosylsphingosine: 1450 nmol/L	Complete resolution of respiratory compromise and hepatomegaly Resolution of cytopenias and reduction (∼36 fold) in plasma glucosylsphingosine	NR
Aries et al., 2022 [19]	Type 2	Clinical manifestations: ichthyosis, moderate neurological abnormalities, particularly a discreet tendency to hyperextend the upper and lower limbs as well as moderate hyperexcitability Muscle tone was elevated. Laboratory values: GCase‐activity: 52.65 pmol/spot^*^20 h Lyso‐GB1: 322,0 ng/mL Chitotriosidase: 2913 nmol/mL/h	Three months after initiation of treatment, neurological examination was normal, and ECGs and echocardiographs displayed normal cardiac anatomy and function during 2 years of treatment. 20‐fold increase in GCase activity, Lyso‐GB1: 673 ng/mL, Chitotriosidase: 42,015 nmol/mL/h	Increased mucus production, high‐pitched screaming and hyperextension in a prone position which improved
Zhang et al., 2022 [20]	Type 1	Clinical manifestations: 1‐year history of oesophageal varices, 34‐year history of liver and spleen enlargement Laboratory values: NR	Decrease in liver stiffness (−55.5%) and portal vein diameter (−41.2%) Increase in GBA activity (+35.5%)	NR
Darling et al., 2021 [33]	Neuronopathic GD with an intermediate form between type 2 and 3 with concomitant features of PD	Clinical manifestations: Psychomotor delay with axial hypotonia, language delay, oculomotor apraxia, splenomegaly, hepatomegaly, hypokinesia, bradykinesia and rest tremor. Laboratory values: Thrombocytes: 70.000–90.000/mm^3^ ALAT: 43–120 UI/L ASAT: 77–223 UI/L Chitotriosidase: 378–998 nmol/mL/hour Bone marrow aspiration study showed foamy cells	The neurological manifestations of one patient worsened	NR
Ramadža et al., 2021 [18]	Type 3	Sibling 1: Clinical manifestations: tricuspid atresia type 1B, hepatosplenomegaly, convergent concomitant strabismus, seizures, EEG showed multifocal discharges, delayed growth, stridor and oculomotor apraxia Laboratory values: Thrombocytes: 119 × 109/L ALT: 65 U/L AST: 28 U/L GCase activity: 0.9 nmol/mg protein/h Chitotriosidase > 12,000 mU/mL Lyso‐Gb1: 418 ng/mL Sibling 2: Clinical manifestations: mild splenomegaly Laboratory values: Thrombocytes: 135 × 10^9^/L	Sibling 1: Better stamina, less expressed oculomotor apraxia, dysarthria and stridor, seizure‐free Chitotriosidase normal a year after treatment, Lyso‐Gb1: 44.9 ng/mL Sibling 2: No clinical manifestations of GD Chitotriosidase normal 3 months after treatment, Lyso‐Gb1: 0.1 ng/mL	Increased mucus production and soft stools
Ciana et al., 2020 [32]	Type 3 (both patients)	Patient 1: Clinical manifestations: myoclonic epilepsy and pathological saccadic movements Laboratory values: Chitotriosidase: 247.5 nmol/mL/h Lyso‐Gb1: 11.4 ng/mL Patient 2: Clinical manifestations: drug‐resistant epilepsy, right eye retinal detachment, secondary glaucoma and severe myopia Laboratory values: Chitotriosidase: 317.92 nmol/mL/h	Patient 1: Frequency of seizures decreased Chitotriosidase: 37.2 nmol/mL/h Lyso‐Gb1: 3.8 ng/mL Patient 2: No change in clinical manifestations and laboratory values of note	Mild abdominal discomfort
Chu et al., 2020 [31]	Type 3	Clinical manifestations: trunk rigidity, mild horizontal gaze palsy and strabismus Laboratory values: GCase activity: 0.47 μM/h Chitotriosidase: 577.62 nmol/Ml, Platelets: 174,000/μL Lyso‐GB1: 55.53 ng/mL	Haemoglobin levels, platelet counts and liver/spleen size all remained stable Lyso‐GB1: 32.7 ng/mL	NR
Jiang et al., 2020 [16]	Type 1	Clinical manifestations: severe pain in the bilateral lower extremities, mild abdominal distention, hepatomegaly, splenomegaly and bilateral femoral head aseptic necrosis Laboratory values: Chitotriosidase: 13,579.6 nmol/h mL WBC: 6 × 10^9^/L RBC: 3.61 × 10^12^/L Hgb: 94 g/L Platelets: 87 × 10^9^/L GCase activity: 1.2 nmol/h/mg protein	Bilateral reshaping of the femoral heads, hepatomegaly and splenomegaly normalised Chitotriosidase: 5235 nmol/h mL WBC: 8.6 × 10^9^/L RBC: 3.82 × 10^12^/L Hgb: 106 g/L Platelets: 140 × 10^9^/L,	None
Pawlinski et al., 2020 [17]	Type 3	Clinical manifestations: growth retardation and splenomegaly Laboratory values: Chitotriosidase: 210 nmol/mL/h	Analgesic effect reported Chitotriosidase: 168 nmol/mL/h	None
Charkhand et al., 2019 [15]	Type 1 (both patients)	Patient 1: Clinical manifestations: refractory epilepsy, wheelchair dependent Laboratory values: Lyso‐Gb1: 35.4 ng/mL GCase activity: 1.62 μmol/h/mg protein Patient 2: Clinical manifestations: refractory epilepsy, progressive ataxia, tremor, wheelchair‐bound and status epilepticus Laboratory values: Lyso‐Gb1: 31 ng/mL GCase activity: 0.6 μmol/h/mg protein	Patient 1: Ataxia improvement Lyso‐Gb1: 10.5 ng/mL Patient 2: Significant ataxia improvement and shorter seizures Lyso‐Gb1: 10 ng/mL	None
Stanescu et al., 2019 [27]	Type 3	Patient 1: Clinical manifestations: seizures and severe psychomotor delay Patient 2: Clinical manifestations: hepatosplenomegaly and seizures	Patient 1: Drop of the biomarkers levels, especially of lyso‐GB1 Slightly improved motor skills Patient 2: Clinical decrease in seizure frequency and intensity with practically normal Lyso GB1 values when the patient was compliant with therapy	NR
Bello and Cortez, 2018 [26]	Type 3	Clinical manifestations: grade IV hepatosplenomegaly. Pale, fussy, hyporexic, with mild breathing difficulty, from the enlarged abdomen, oculomotor apraxia and skeletal manifestations Laboratory values: GCase activity low, anaemia and thrombocytopenia. Bone marrow biopsy showed almost total replacement of normal hematopoiesis, with Gaucher cells.	Improvement of neurologic symptoms Blood counts, including platelets, normalized shortly after starting enzyme replacement therapy. Decreased hepatosplenomegaly Increased GCase activity	NR

Abbreviations: ALAT, alanine aminotransferase; ASAT, aspartate aminotransferase; GCase, Glucocerebrosidase; HgB, Haemoglobin; Lyso‐GB1, glucosylsphingosine; NR, not reported; RBC, red blood cells; WBC, white blood cells.

### 3.3 The effect of Ambroxol on laboratory values

Kim et al. [1] reported on patients with GD and myoclonic epilepsy. With a mean baseline of 5.1 ± 0.6% peripheral leucocytes’ activity compared to normal, 21 and 27 mg/kg/day Ambroxol led to activities 9.8 ± 3.2% (*p* = 0.042) and 13.7 ± 4.4% (*p* = 0.02) of the mean normal activity, respectively. There were no significant changes in the haematological profile nor bone densitometry findings following 4.5 years of treatment with Ambroxol.

Zimran et al. [35] reported on a 6‐month follow‐up of 12 patients with type 1 GD treated with Ambroxol. This resulted in minimal changes in body weight, haemoglobin levels, liver volume and chitotriosidase activity. The mean platelet count increased from 84.4 to 92.4 × 10^3^/mm^3^ and the mean spleen volume decreased from 1616 to 1357 cm^3^. Whether changes from baseline were statistically significant was not reported, with outcome measures globally unchanged overall after 6 months.

Istaiti et al. [28] treated 40 type 1 GD patients with Ambroxol. Of these, 28 had 3 years of sub‐optimal response to ERT or SRT (platelet count < 100 × 103/L, lumbar spine bone density T‐score < −2.0 and/or LysoGb1 > 200 ng/mL), and 12 were naïve to treatment. Sixteen completed a 12‐month treatment course. Reasons for withdrawal included adverse events (*n* = 12), coronavirus disease 2019 (*n* = 7), the inability to swallow Ambroxol capsules (*n* = 3), receiving a new diagnosis of PD, being advised to receive a higher dose of Ambroxol than that which was administered as part of the trial (*n* = 1), and participation in another clinical trial (*n* = 1). Five patients (31.2%) achieved greater than 20% improvement in platelet count and three patients (18.9%) had a 20% or greater reduction in Lyso‐Gb1 at the end of treatment.

Narita et al. [5] reported on five patients with GD. Glucocerebrosidase activity in lymphocytes increased by 171.1% (*p* = 0.03) and reached levels observed in carriers or control subjects. Cerebrospinal fluid glucosylsphingosine levels fell by 25.7% (*p* = 0.03) after therapy. Mullin et al. [29] administered Ambroxol to 18 patients with GD‐associated PD. At 186 of follow‐up, only improvements in cerebrospinal fluid GCase activity, α‐Synuclein, GCase protein level and blood Tau were statistically significant. Zhan et al. [30] reported statistically significant improvements in haemoglobin concentration, spleen and liver volume, Chitotriosidase activity and Glucosylsphingosine level were noted in 28 patients following a mean of 2.6 years of treatment with Ambroxol. Changes in laboratory values were not reported in one case report [33]. The remaining reports reported improvements in laboratory values (Table 5).

## 4 DISCUSSION

Multiple laboratory studies have described the mechanisms by which Ambroxol may provide symptomatic relief in patients with GD. These include increasing GCase activity and reducing hexosylsphingosine concentration [36], increasing mutant β‐glucosidase activity [37], and ameliorating the unfolded protein response [38]. Though to varying degrees, all studies reported improvements in clinical parameters and/or laboratory values, with the exception of two case reports [32, 33]. However, the evidence was low in quality, with methodological limitations pertaining to the low level of evidence and concerns regarding the risk of bias. In addition, only 182 patients were included. Caution should therefore be placed when interpreting these findings.

A single type of GD comprised the entire cohort in seven case series, with the remaining two reporting multiple types (with only one reporting outcomes for these separately [28]). Three case series reported on type 1 GD [28, 35, 39]. However, only two reported clinical outcomes. These found 36% [39] and 55% [28] of patients reported symptomatic relief. The remaining study reported on changes in laboratory values rather than clinical parameters and did not state whether these were statistically significant. Further research evaluating clinical outcomes following Ambroxol for type 1 GD is required. Rates of adverse events in type 1 patients ranged from 8.5% to 35%. No severe adverse events were reported. In addition, three case reports pertaining to type 1 GD reported a lack of adverse events [15, 16, 20]. This suggests Ambroxol could be a safe therapy for type 1 GD.

Of the two case series reporting on type 2 GD [3, 25], one found a 22% treatment response rate, whereas the other stated mortality within 6 months was 40%. In addition, a case report found complete resolution of respiratory compromise, hepatomegaly, and cytopaenia following Ambroxol therapy in treatment with type 2 GD [34]. These did not report on Ambroxol's safety profile, with further research required to ascertain this in type 2 GD.

Kim et al. [1] reported on type 3 GD exclusively and found improvements in symptom scores. However, the statistical significance of these changes was not reported. Despite a 50% complication rate, no severe adverse events were reported. Istaiti et al. [28] noted that 29% of patients with type 3 GD reported neurological improvements. In addition, of six case reports pertaining to type 3 GD, five found symptomatic improvement [17, 18, 26, 27, 31].

Current evidence suggests Ambroxol may be used for the treatment of neurological manifestations of GD, though varying response rates are to be expected (29%–100%) [1, 5, 8]. Lal et al. [3] reported statistically significant improvements in symptom scores in patients with PD, whereas Istaiti et al. [28] noted one in three patients with PD reported symptomatic improvement. Darling et al. [33] reported a patient with concomitant features of PD experienced worsening of neurological manifestation. Though this may suggest Ambroxol may not be useful for the treatment of GD‐associated PD, further research is required.

Only a single case series and two case reports (comprising two patients each) reported Ambroxol's effects on epilepsy, with two of four patients with myoclonic epilepsy exhibiting reductions in number of seizures experienced [1], and each case report describing that only one of each of their two patients experienced a decrease in seizure frequency [27, 32]. Further research evaluating Ambroxol's ability to treat epilepsy is needed, given its impact on patients’ quality of life and prognosis. ERTs such as imiglucerase do not cross the blood‐brain barrier, which leads to neurologic manifestations of GD2 and GD3 being refractory to this treatment [40]. SRTs such as miglustat do not appear to have significant benefits on the neurological manifestations of GD3 [41]. Ambroxol could be considered for the treatment of patients with neurological symptoms of GD due to response rates ranging from 29% and 100%, and its availability over the counter. It may yield improvements in neurological symptoms in cases in which ERT and SRT are unable to achieve these due to their mechanism of action. In fact, Aries et al. [19] presented the first report of a patient with type 2 GD achieving developmental milestones at 3 years of age as a result of Ambroxol therapy (25 mg/kg/day). Though this consists of a single case, it may provide an incentive to conduct further work on Ambroxol as a means to foster neurological development in patients with GD. However, the low sample size and level of evidence of the studies included in this review must be taken into account when assessing the efficacy of Ambroxol for neurological manifestations of GD. Further work on larger sample sizes should be conducted to ascertain the validity of these findings.

Variable response rates to Ambroxol were reported, with different degrees of improvement observed. This could be attributed to the heterogeneous nature of Gaucher disease, with diverse presentations leading to varied responses. Treatment strategy must therefore be adapted according to patient presentation to optimize outcomes [2]. Treatment guidelines recommend early multi‐disciplinary team involvement for cases of GD [8]. Clinical judgment is advised to guide decisions regarding the use of Ambroxol for the treatment of GD, taking patient presentation and previously unsuccessful therapies into account. Ambroxol was generally well tolerated, with rates of adverse events ranging from 8.5% to 60%. However, despite the high complication rate, adverse events were mild and reversible, with respiratory difficulty due to an increase in mucus production and uricaemia among the most severe complications. In addition, the study reporting a 60% complication rate consisted of five patients only [5], whereas the study reporting a 50% complication rate consisted of four patients [1]. The low sample size of these studies could explain the high rate of adverse events observed. Overall response rates ranged from 22% to 61%, and improvements in laboratory values and symptom scores were noted. Neurological response rates ranged from 29% to 100%. Therefore, clinicians may consider the counter‐use of Ambroxol for symptomatic relief in patients with incurable forms of GD, balancing potential benefits and the effect of adverse events on patients’ quality of life. Ambroxol may achieve more notable clinical effects during the presymptomatic or early disease period. Therefore, early intervention is desirable [5]. Even though high‐dose Ambroxol may provide symptomatic relief in patients with GD, not much is known about its long‐term effects. Careful observation must take place to ensure continued patient safety [5], particularly because symptomatic improvement may not be achieved in the short term [1]. Follow‐up duration of the studies included in this review ranged from 6 months to 7 years, exemplifying the long treatment course. Informed patient discussions are required to maximize compliance with the long duration of Ambroxol therapy for GD. Clinicians may encounter barriers to its administration [28]. First, there is likely to be a lack of reimbursement, since it is not a registered therapy. Second, despite Ambroxol being a relatively inexpensive drug, many families in developing countries may not be able to afford it. Third, doses required for symptomatic relief are much higher than the concentrations available over the counter. Therefore, adults need to take a large number of capsules daily, and children need to drink a large amount of syrup, which may affect patient compliance.

Current evidence has limitations which must be improved to increase the understanding of Ambroxol as a therapeutic approach for GD. First, the studies identified carried a low level of evidence with concerns regarding their risk of bias, with a total of only 182 patients included. All studies were case series (three of which comprised less than five patients) or case reports. Though they are feasible study designs to evaluate rare diseases like GD, they are prone to publication and selection bias. Secondly, there was significant study heterogeneity pertaining to differing study designs, differing treatment regimens, patient characteristics and outcomes evaluated. This limits the ability to extrapolate the studies’ results and hinders the performance of future meta‐analyses on the subject. Thirdly, no studies comparing Ambroxol to other treatment regimens have been identified. Such work is required to determine whether Ambroxol provides an additional benefit over ERT and SRT for GD, considering the high costs and adverse events associated with these conventional therapies [13]. In addition, there have been no clinical trials conducted for the registration of Ambroxol as a treatment for GD. Considering Ambroxol is a relatively inexpensive over‐the‐counter drug, conducting well‐designed prospective clinical trials is unlikely; explaining why these have never been performed [13]. International collaborations may help define the optimal doses and mode of administration of Ambroxol for the treatment of GD. Namely, the recently established global neuronopathic Gaucher disease registry (GARDIAN) [42] should encourage contributing clinicians to report on their experience with Ambroxol to make progress towards an international consensus. No studies performing cost‐effectiveness analyses have been performed. Though Ambroxol can be purchased over the counter, other prescription‐only medications may facilitate more cost‐effective treatment of GD. Fourthly, a single study treated type 1 GD patients with previous sub‐optimal responses to ERT/SRT [39], and it reported on laboratory values rather than the effect on symptoms. Further research on Ambroxol for the treatment of type 1 GD with poor response to ERT/SRT is required. Finally, though three studies performed in‐vitro analyses of responses to Ambroxol [1, 5, 29], no studies performed in‐vitro tests to predict response to treatment. Such work could provide clinicians with tools to determine which patients would benefit from Ambroxol therapy. Regarding monitoring treatment response, biomarkers used should be specific to GD, be widely available and highly sensitive, and be directly involved in the pathological pathway of GD [43].

## 5 CONCLUSION

Current evidence suggests Ambroxol may be considered for the treatment of neurological manifestations of GD. However, varying degrees of symptomatic improvement in the treatment of GD were noted. This could be attributed to its heterogeneous nature, with diverse patient presentations leading to differing responses to therapy. Multidisciplinary team input and clinical judgement are advised to provide personalized treatment of this complex and multi‐faceted condition.

## AUTHOR CONTRIBUTIONS

**Diego Agustín Abelleyra Lastoria**: conceptualization; database search; data extraction; risk of bias assessment and writing—original manuscript preparation. **Simranjeet Grewal**: database search and risk of bias assessment. **Derralynn Hughes**: conceptualization and writing—manuscript editing and supervision.

## CONFLICT OF INTEREST STATEMENT

DH has received honoraria for speaking and advisory boards from Sanofi, Takeda and Freeline administered through UCL consultants.

## FUNDING INFORMATION

None.

## ETHICS STATEMENT

The authors have confirmed ethical approval statement is not needed for this submission.

## PATIENT CONSENT STATEMENT

The authors have confirmed patient consent statement is not needed for this submission.

## CLINICAL TRIAL REGISTRATION

The authors have confirmed clinical trial registration is not needed for this submission.

## Data Availability

Not applicable.
